# MiR-370 sensitizes chronic myeloid leukemia K562 cells to homoharringtonine by targeting Forkhead box M1

**DOI:** 10.1186/1479-5876-11-265

**Published:** 2013-10-23

**Authors:** MinRan Zhou, JiPing Zeng, XiaoMing Wang, Qing Guo, Tao Huang, HaiYu Shen, Yue Fu, LiXiang Wang, JiHui Jia, ChunYan Chen

**Affiliations:** 1 Department of Hematology, Qilu Hospital, Shandong University School of Medicine, Jinan, P.R. China; 2 Department of Microbiology/Key Laboratory for Experimental Teratology of Chinese Ministry of Education, Shandong University School of Medicine, Jinan, P.R. China; 3 Department of Biochemistry, Shandong University School of Medicine, Jinan, P.R. China; 4 Department of Pharmacology, Shandong University School of Medicine, Jinan, P.R. China; 5 Department of Gastrointestinal Surgery, Jinan Central Hospital, Shandong University School of Medicine, Jinan, P.R. China

**Keywords:** miR-370, HHT, Cellular apoptosis, Chronic myeloid leukemia

## Abstract

**Background:**

Homoharringtonine (HHT) is a kind of cephalotaxus alkaloid used in traditional Chinese medicine. Although HHT has been successfully used as a therapeutic agent for leukemia, the drug resistance and toxicity are major concerns. MicroRNAs (miRNAs) have been identified to modulate cellular sensitivity to anticancer drugs. We examined the synergistic action between miR-370 and HHT *in vitro* and *in vivo*.

**Methods:**

The synergistic action between miR-370 and HHT was examined by flow cytometry. The effect of HHT on miR-370 expression was determined by quantitative RT-PCR (qRT-PCR). The expression of miR-370 and Forkhead box M1 (FoxM1) in 23 patients with newly diagnosed chronic-phase chronic myeloid leukemia (CML-CP) and 10 patients with blast-crisis CML (CML-BP) as well as miR-370–targeted FoxM1 was determined by qRT-PCR and western blot analysis.

**Results:**

Ectopic expression of miR-370 sensitized the CML K562 cell line to HHT by targeting FoxM1, the major regulator in cell proliferation and apoptosis. miR-370 significantly promoted HHT-mediated cell apoptosis and miR-370 and HHT cooperated in affecting FoxM1 expression. As well, miR-370 was moderately upregulated after HHT treatment in K562 cells. In addition, the expression of miR-370 was significantly reduced in CML patients as compared with healthy controls. Furthermore, the expression of miR-370 was lower in CML-BP than CML-CP patients.

**Conclusions:**

MiR-370 sensitized K562 cells to HHT by inducing apoptosis in part by downregulation of FoxM1 expression. These findings may provide further information for CML treatment with HHT.

## Background

Homoharringtonine (HHT), a plant alkaloid, is a traditional Chinese medicine that has been successfully used for leukemia treatment [1]. In the 1970s, a mixture of HHT and harringtonine (HT) was first used to treat acute myeloid leukemia (AML) and chronic myeloid leukemia (CML) in China [2,3]. HHT inhibits G1 and G2 protein synthesis, induces cell differentiation and promotes cell apoptosis [4-6]. HHT was also effective in the treatment of CML after interferon α (IFN-α) failure [7].

HHT has synergistic activity with imatinib in imatinib-resistant cell lines and primary cells from patients with CML in blast crisis (CML-BP) [8]. Phase I and II studies in the United States confirmed the clinical efficacy of HHT in CML but documented a high incidence of cardiovascular complications by intravenous administration [9]. Studies *in vitro* also revealed a cooperative action between HHT, Ara-C and IFN-α [10].

MicroRNAs (miRNAs) are small non-coding RNAs that regulate gene expression by directly binding to the 3′ untranslated regions (3′ UTR) of the target gene mRNA, inducing translational inhibition or degradation [11,12]. miRNAs are misregulated in human cancers [12] and are involved in several biological processes such as development, proliferation, differentiation, and apoptosis [13]. Recently, miRNAs were found active in the chemosensitivity and chemoresistance of human cancer cells [14,15]. For example, the inhibition of miR-21 sensitized K562 cells to arsenic trioxide [16]. miR-370 is downregulated in gastric cancer [17], colorectal cancer [18] and malignant human cholangiocytes [19]. Our group also certified that miR-370 is downregulated in AML and is involved in cell proliferation by directly targeting the 3′ UTR of Forkhead box M1 (FoxM1), the key positive transcriptional factor in the cell cycle and found overexpressed in many tumor types [17,20]. However, the role of miR-370 in the chemosensitivity of leukemic cells is unknown.

We aimed to define whether miR-370 has a synergistic effect with HHT via FoxM1 in CML. We investigated a lower dose of HHT to reduce its toxicity and maintain its function.

## Method

### Patients and bone marrow samples

Patient bone marrow samples were collected between June 2009 and December 2012 at the Department of Hematology, Qilu Hospital, Shandong University School of Medicine, Jinan, China. Bone marrow samples were obtained from patients with newly diagnosed CML in the chronic phase (CML-CP, n = 23) and blast crisis (CML-BP, n = 10). Negative control samples came from 14 healthy volunteers. Mononuclear cells were isolated from the samples by Ficoll-Hypaque density gradient centrifugation, then stored at -80°C. The study was approved by the Ethics Committee of Shandong University School of Medicine.

### Cell culture and transfection

The human CML cell line K562 was cultured at 37°C, 95% air and 5% CO_2_ in RPMI 1640 containing 10% heat-inactivated fetal bovine serum (FBS) without antibiotics (Gibco, Carlsbad, CA, USA). Cells were cultured on 6-well plates for 18 to 24 h before experiments. K562 cells were tranfected with miR-370 mimics (miR10000722-1-5) and inhibitor (miR20000722-1; Ribobio, Guangzhou, China) by use of Lipofectamine 2000 (Invitrogen, Carlsbad, CA, USA), then 6 h later transfected with HHT (0.015 μM). K562 cells were tranfected with FoxM1 siRNA or FoxM1 overexpression plasmid with Lipofectamine 2000 (Invitrogen, Carlsbad, CA, USA)for 72 h. FoxM1 siRNA was designed and sythesized by Invitrogen. The sequence for the FoxM1 siRNA was 5′-GACAACUGUCAAGUGUACCACUCUU-3′. FoxM1 overexpression plasmid was constructed by our group and the primer sequences were 5′ the primer sequences were 5′-GAAGATCTTAACCATGAAAACTAGCCCCCG-3′(Forward) and 5′ -CGGAATTCGCTACTGTAGCTCAGGAATAAA-3′(Reverse).

### RNA extraction and quantitative RT-PCR

The total RNA in human BM sample and K562 cells was extracted by use of Trizol (Invitrogen, Carlsbad, CA, USA). The expression of miR-370 was detected by quantitative RT-PCR (qRT-PCR) with the TaqMan miRNA assay kit (Applied Biosystems, Foster City, CA, USA) and U6 snRNA used as a control. In summary, total RNAs were used for RT with specific primers, with the reaction mixtures incubated at 16°C for 30 min, 42°C for 30 min and 85°C for 5 min. Then RT products were used as templates for real time-PCR. PCR cycles were an initial denaturation at 95°C for 10 min. Then the reaction was repeated for 40 cycles of denaturing at 95°C for 10 s, annealing and synthesis at 60°C for 60 s. qRT-PCR involved use of the ABI7500 sequence detector (Applied Biosystems, Foster City, CA, USA). The level of miR-370 expression was normalized by U6 snRNA. The mRNA level of FoxM1 was determined by RT and SYBR-Green real-time PCR assay. cDNA was synthesized with a random primer and MMLV reverse transcriptase (Fermentas, Canada). Real-time PCR involved the ABI7500 sequence detector (Applied Biosystems, Foster City, CA, USA). The PCR primer sequences were for FoxM1, 5′-TGCAGCTAGGATGTGAATCTTC-3′ (Forward) and 5′-GGAGCCCAGTCCATCAGAACT-3′ (Reverse); β-actin: 5′-AGTTGCGTTACACCCTTTCTTG-3′ (Forward) and 5′-CACCTTCACCGTTCCAGTTTT-3′ (Reverse). FoxM1 mRNAs were normalized to β-actin expression. Expression was calculated as the change relative to the control (2^-∆∆Ct^).

### Western blot analysis

The cells were lyzed in protein lysis buffer in the presence of proteinase inhibitor (Biocolor BioScience & Technology, Shanghai). Proteins were separated by SDS-PAGE and transferred to PVDF membranes, which were probed with primary antibodies against FoxM1 (1:500, Santa Cruz Biotechnology, Santa Cruz, CA, USA) and β-actin (1:20000, Sigma) for 2 h under room temperature followed by horseradish peroxidase-labeled goat-anti-rabbit IgG (1:6000, Santa Cruz Biotechnology) for 2 h. The signals were detected by enhanced chemiluminescence. β-actin acted as a loading control.

### Flow cytometry

K562 cells were seeded in 6-well plates for treatment with miR-370 and/or HHT (0.015 μM) for various times. Then 10^6^ cells were harvested for each group and washed twice with PBS. The cells were double-stained with FITC-conjugated Annexin V and propidium iodide (PI). Apoptosis and necrosis were analyzed by quadrant statistics. Data are shown as the percentage of apoptotic cells.

### Statistical analysis

All the experiments were carried out in triplicate. Data are expressed as mean ± SEM. Differences were calculated by two-tailed Student’s *t* test or one-way ANOVA for experiments with more than 2 subgroups by use of SPSS 13.0 (SPSS Inc., Chicago, IL). Statistical significance was defined as *P* < 0.05.

## Results

### Upregulation of miR-370 sensitized K562 cells to HHT

**Figure 1:**
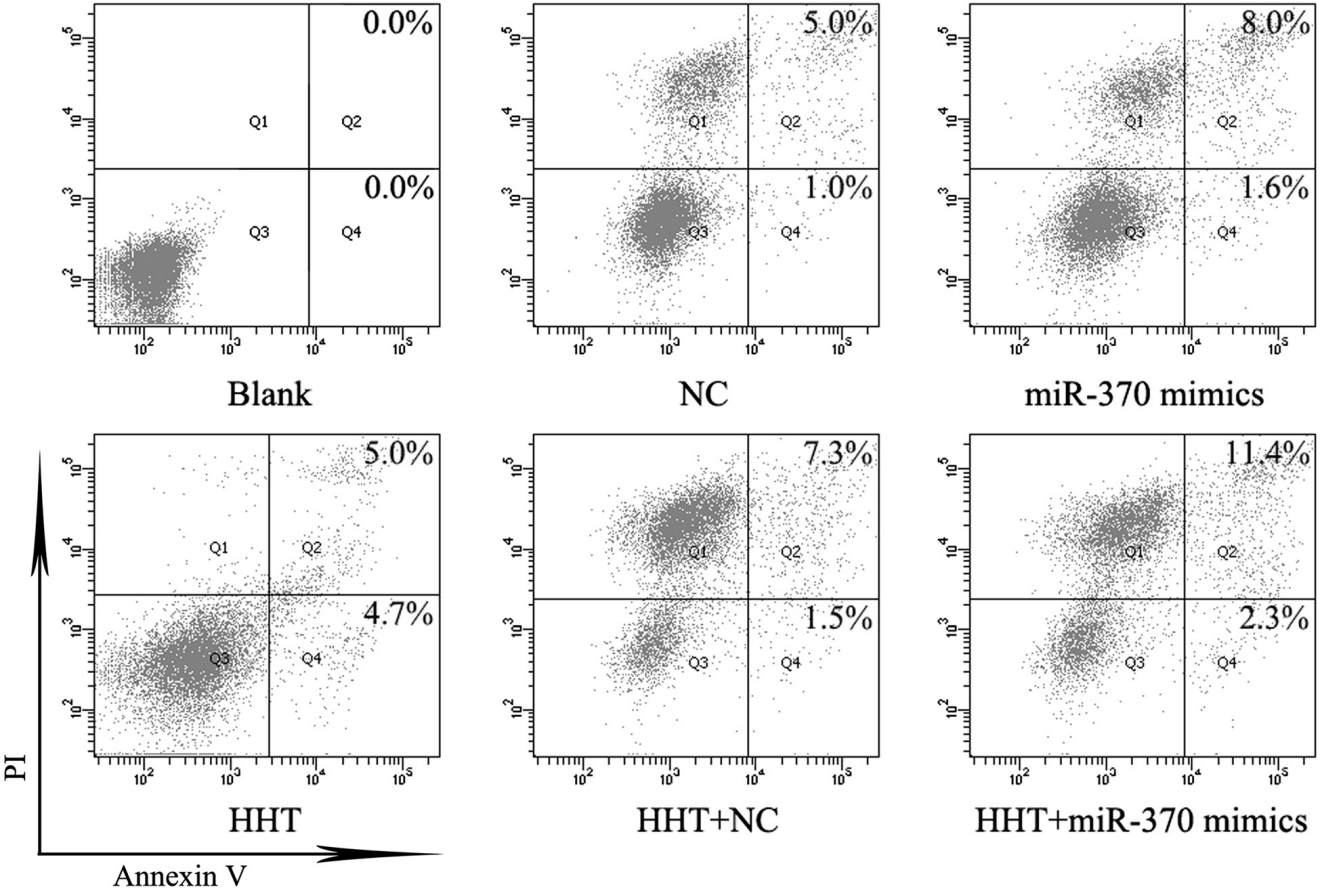
**MiR-370 enhanced HHT-induced cell apoptosis in K562 cells.** Cells were harvested 72 h after transfection of miR-370 mimics and incubation with 0.015 μM HHT or both. NC represents random oligonucleotides for the negative control of miR-370 mimics. Flow cytometry of K562 cells by double-staining with propidium iodide (PI) and Annexin V. The proportions of apoptotic cells are in the upper right corners of Q2 and Q4 cells. Q2 is late apoptosis and Q4 early apoptosis. Results are from 3 independent experiments.

MiR-370 mimics was transfected into K562 cells alone or with 0.015 μM HHT after 6 h. According to MTT assay of K562 cell proliferation, IC50 values of HHT was determined and 0.015 μM HHT was selected (Additional file 1: Figure S1). After 72 h incubation, the proportion of apoptotic K562 cells was detected by flow cytometry by double-staining with PI and Annexin V. Both miR-370 mimics and HHT induced cell apoptosis (Figure 1) (*P* < 0.05, Additional file 2: Figure S2). More importantly, miR-370 promoted HHT-induced cell apoptosis (*P* < 0.05, Additional file 2: Figure S2).

**Figure 2:**
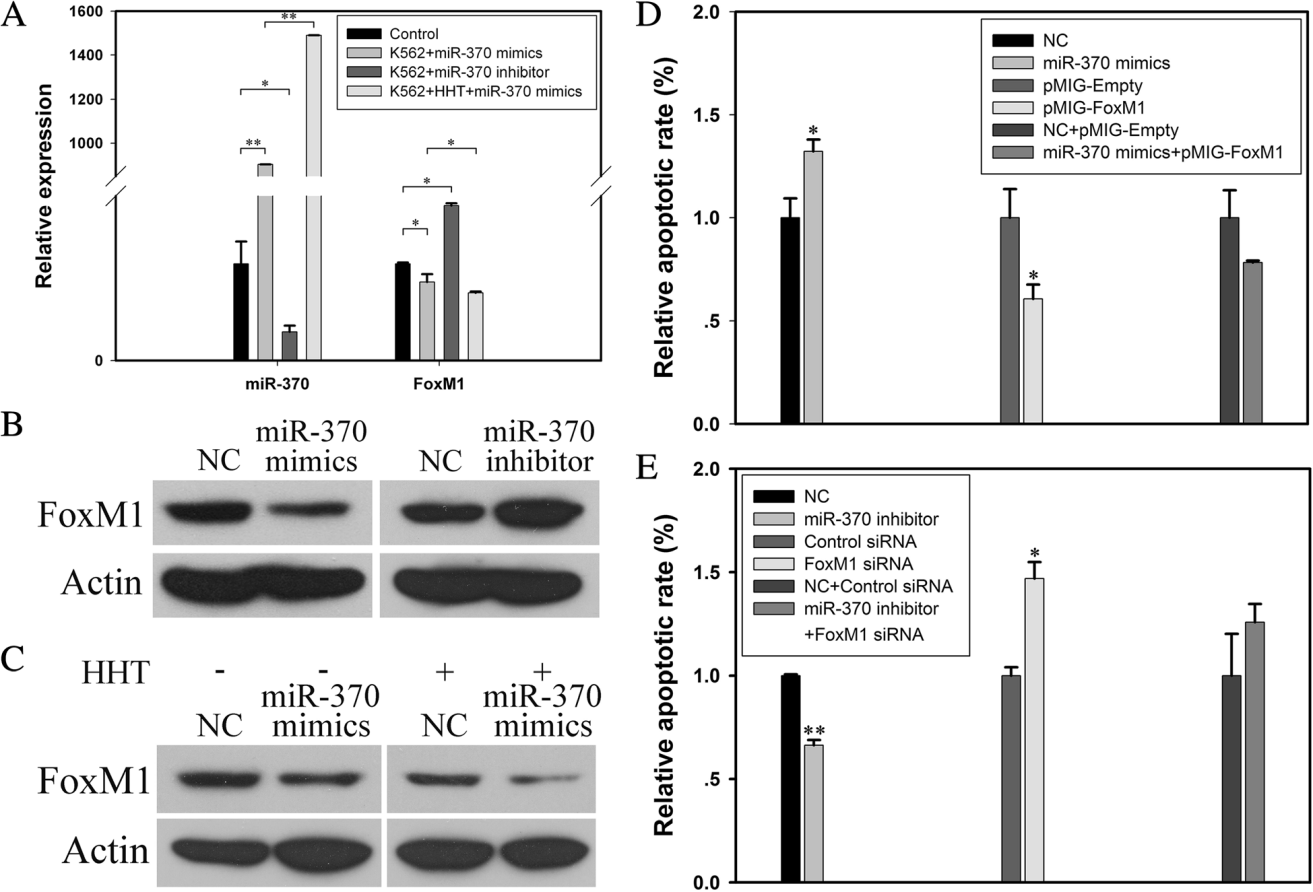
**MiR-370 enhanced HHT-induced apoptosis dependent in part on FoxM1 in K562 cells. (A)** The mRNA expression of FoxM1 was regulated by miR-370 mimics or inhibitor at the transcriptional level. Quantitative RT-PCR (qRT-PCR) analysis of miR-370 and FoxM1 and the loading control U6 snRNA. The time for transfection of miR-370 and incubation of 0.015 μM HHT is 72 h. Data are mean ± SEM of 3 independent experiments. **(B)** Western blot analysis of FoxM1 protein level after treatment with miR-370 mimics and inhibitor. β-actin was a loading control. **(C)** after transfection with miR-370 mimics with or without HHT. **(D)** Quantitative flow cytometry of the apoptosis of K562 cells. Cells were harvested 72 h after transfection of miR-370 mimics or FoxM1 overexpression plasmid. The results are from 3 independent experiments. **(E)** Cells were harvested 72 h after transfection of miR-370 inhibitor or FoxM1 special siRNA (5 μM). Quantitative flow cytometry of the apoptosis of K562 cells. The results are from 3 independent experiments. *P < 0.05, **P < 0.01.

The mRNA level of miR-370 in K562 cells was significantly increased with the transfection of miR-370 mimics as compared with the control (*P* < 0.01) (Figure 2A). The expression of miR-370 was greater with HTT + miR-370 mimics as compared with miR-370 mimics alone (*P* < 0.01), which suggested that the upregulation of miR-370 sensitized K562 cells to HHT for apoptosis and the possible effect of HTT on miR-370 expression.

### Increased sensitivity to HHT with upregulation of miR-370 was partially attributed to FoxM1 downregulation

To further determine the correlation between HHT, miR-370 and FoxM1 in the CML K562 cell line, we checked the expression of FoxM1 in cells. After transfection with miR-370 mimics or inhibitor, the expression of miR-370 was overexpressed and downregulated, respectively (Figure 2A). As well, the mRNA and protein levels of FoxM1 were inhibited with miR-370 mimics and increased with miR-370 inhibitor, so the expression of miR-370 was negatively related to that of FoxM1 in K562 cells (Figure 2A and 2B). Meanwhile, the expression of FoxM1 was further inhibited with HHT + miR-370 mimics as compared with miR-370 mimics alone (*P* < 0.05, Figure 2A). The protein expression of FoxM1 was inhibited with HHT + miR-370 mimics as compared with HHT + NC and miR-370 mimics alone (Figure 2C). Thus, FoxM1 had a role in increased sensitivity to HHT with upregulation of miR-370.

To further identify the role of FoxM1, we checked its function in cell apoptosis induced by miR-370. After transfection with miR-370 mimics or FoxM1 siRNA, both miR-370 mimics and FoxM1 siRNA induced cell apoptosis (*P* < 0.05, Figure 2D, 2E). Otherwise, miR-370 inhibitor and FoxM1 overexpression plasmid inhibited cell apoptosis (*P* < 0.01, Figure 2E; *P* < 0.05, Figure 2D). When FoxM1 overexpression plasmid was transfected into K562 cells with miR-370 mimics to reverse the expression of FoxM1, the apoptosis was partially reversed. However, the FoxM1 siRNA that inhibited the miR-370-inhibitor–induced overexpression of FoxM1 neutralized the inhibited apoptosis induced by anti-miR-370 treatment.

### HHT mediated the upregulation of miR-370 in K562 cells

**Figure 3:**
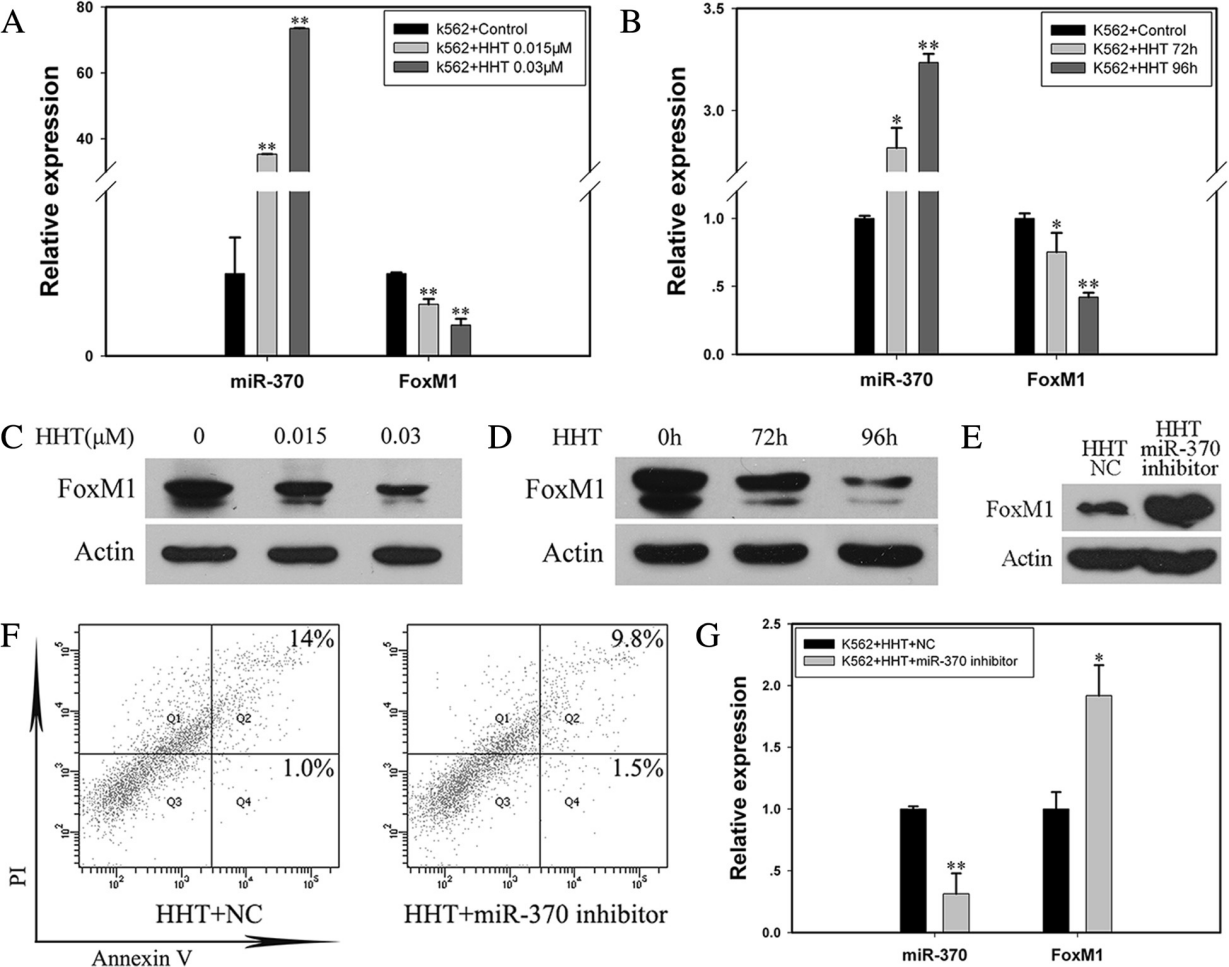
**HHT regulated mature miR-370 level in K562 cells.** K562 cells were incubated with HHT or PBS and harvested after 72 or 96 h. Real-time PCR analysis of the mRNA expression of mature miR-370 and FoxM1. Western blot analysis of FoxM1 protein level. **(A)** HHT increased the expression of mature miR-370 in a dose-dependent manner. The mRNA level of FoxM1 decreased in K562 cells with HHT incubation, **P < 0.01 *vs* control, Data are mean ± SEM of 3 independent experiments. **(B)** HHT regulated the expression of miR-370 and FoxM1 time-dependently. *P < 0.05, **P < 0.01 *vs* control, Data are mean ± SEM of 3 independent experiments. **(C-D)** Western blot analyis of protein level of FoxM1 after HHT treatment at different concentrations or different times. β-actin was a loading control. **(E)** The protein expression of FoxM1 with incubation of HHT and miR-370 inhibitor. The results were confirmed by 3 independent experiments. **(F)** Anti-miR-370 treatment partially reversed HHT-induced apoptosis. Cells were treated with 0.015 μM HHT + NC or 0.015 μM HHT + miR-370 inhibitor for 72 h. Flow cytometry of K562 cells by double-staining with PI and Annexin V. The proportion of apoptotic cells is shown in the upper right corners of Q2 and Q4 cells. The results are from 3 independent experiments. **(G)** Mature miR-370 expression was downregulated in K562 cells treated with HHT + miR-370 inhibitor, **P < 0.01 *vs* HHT + NC group. FoxM1 mRNA level was upregulated, *P < 0.05 *vs* HHT + NC group. Data are mean ± SEM of 3 independent experiments.

To further investigate the effect of HHT on miR-370 expression, we detected the expression of miR-370 and its target FoxM1 with incubation of HHT in K562 cells. In cells incubated with HHT at different concentrations (0.015 and 0.03 μM) for 72 h, the level of mature miR-370 increased to about 37-fold and 77-fold that of the control (*P* < 0.01; Figure 3A). FoxM1 mRNA and protein expression was dose-dependently downregulated (*P* < 0.01; Figure 3A, 3C). Furthermore, after K562 cells were incubated with HHT for 72 and 96 h, the level of miR-370 was upregulated (*P* < 0.05; *P* < 0.01), which was accompanied by the inhibition of FoxM1 mRNA and protein expression (*P* < 0.05; *P* < 0.01, Figure 3B, 3D). Therefore, HHT mediated the upregulation of miR-370 in K562 cells.

To further detect the importance of miR-370 in HHT-induced apoptosis, miR-370 inhibitor was transfected into K562 cells with 0.015 μM HHT for 72 h. The miR-370 inhibitor partially reversed HHT-induced cell apoptosis as compared with the control (Figure 3F) (*P* < 0.05, Additional file 3: Figure S3). MiR-370 expression was inhibited and FoxM1 expression was upregulated (Figure 3E, 3G). Therefore, the HHT–miR-370–FoxM1 axis might be a new regulatory mechanism in HHT-induced apoptosis.

### Misregulation of miR-370 and FoxM1 in bone marrow from CML-CP and CML-BP patients

**Figure 4:**
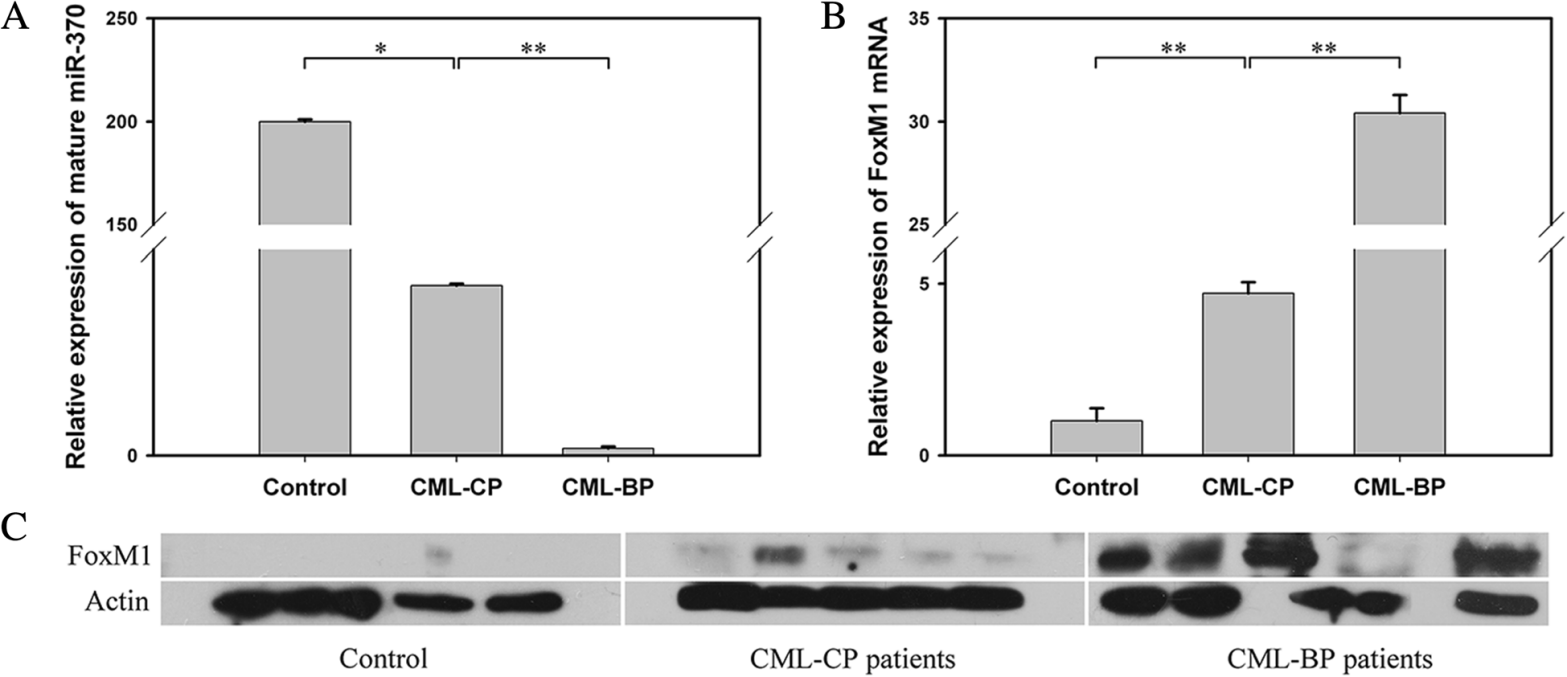
**The expression of miR-370 and FoxM1 in CML-CP and CML-BP patients. (A)** Real-time PCR analysis of the relative expression of mature miR-370 in 23 CML-CP patients, 10 CML-BP patients and 14 healthy controls. *P < 0.05 *vs* control and **P < 0.01 *vs* CML-CP. Data are mean ± SEM. **(B, C)** qRT-PCR and western blot analysis of FoxM1 mRNA and protein levels. **P < 0.01 *vs* control and **P < 0.01 *vs* CML-CP. Data are mean ± SEM. The results were confirmed by 3 independent experiments.

MiR-370 expression was analyzed in bone marrow samples from 23 patients with newly diagnosed CML-CP and 10 with CML-BP. The clinical characteristics of CML patients are in Additional file 4: Table S1. The level of miR-370 was lower in CML patients than healthy controls. Furthermore, miR-370 expression was lower in CML-BP than CML-CP patients (Figure 4A).

The mRNA level of FoxM1 was higher in patients with CML-BP than CML-CP, with the expression lowest in healthy controls (Figure 4B), which showed the negative association with miR-370 expression. The FoxM1 protein expression findings were consistent with the mRNA findings, for lowest level in healthy controls, higher in CML-CP patients and highest in CML-BP patients (Figure 4C).

## Discussion

HHT is a traditional Chinese medicine that has been successfully used for treatment of leukemia [1]. We found that miR-370, which directly targets FoxM1, could sensitize K562 cells to HHT by inducing cell apoptosis, which may give hope for miRNA-based CML therapy with less drug toxicity.

MiRNAs are endogenous substances that translationally inhibit or degrade target gene mRNA by binding to the 3′ UTR of target gene mRNA. Many studies have shown that miR-370 is commonly deregulated in multiple human tumors and implicated in various aspects of tumors, including growth, metastasis and senescence [18-22]. Our group found that miR-370 is involved in AML and *Helicobacter pylori*-induced gastric carcinogenesis by directly targeting FoxM1 [17,20]. In this research, we found that ectopic expression of miR-370 induced apoptosis in the CML cell line K562. More important, miR-370 mimics could improve HHT-induced apoptosis. HHT plays an important role in antitumor therapy by inducing apoptosis [23]. Recent research also showed that HHT was effective when combined with other agents for its cardiotoxicity at relative high concentration [24]. The combination of HHT and miR-370 shows a new way to induce apoptosis in CML K562 cells with less concentration of HHT and therefore fewer side effects. Considering the characteristic of miRNAs in human bodies, this combination of HHT and miR-370 might have clinical value.

To assess the role of abnormally expressed miRNA in human cancer and develop miRNA-based gene therapy, target genes of miRNAs must be identified. Increasing evidence has shown that miR-370 regulates a number of target genes, including Wilms tumor gene on the X chromosome [25], insulin receptor substrate 1 [22], Forkhead box protein O1 [26] and FoxM1 in AML by our group [20]. FoxM1 is the master positive regulator of the cell cycle and is related to cell proliferation, cell cycle progression and apoptosis [27-34]. Furthermore, recent studies suggest that FoxM1 mediates chemoresistance. For example, overexpression of FoxM1 partially protected cancer cells against thiazole antibiotic-mediated cell death [34] and enhanced hepatoma cell resistance to TNF-α–induced apoptosis [30]. FoxM1 knockdown sensitized cancer cells to apoptotic cell death induced by proteasome inhibitors such as MG-132, bortezomib and thiostrepton [35]. Inhibition of FoxM1, combined with oxaliplatin treatment, significantly promoted the senescence of hepatocellular carcinoma cells [36]. Here, we confirmed that FoxM1, as a target gene of miR-370, partially mediated the chemosensitivity of K562 cells to HHT. FoxM1 overexpression reversed cell apoptosis induced by miR-370 mimics in part, and FoxM1 siRNA neutralized the inhibition of apoptosis induced by miR-370 inhibitor. The increased sensitivity of K562 cells to HHT-induced apoptosis, which resulted from ectopic expression of miR-370, was at least in part related to FoxM1.

We also found that HHT + miR-370 mimics upregulated the expression of miR-370 to a higher level as compared with miR-370 mimics alone. We further checked the mechanism among HHT, miR-370 and FoxM1. HHT upregulated the level of mature miR-370 time- and dose-dependently, and anti-miR-370 treatment reversed HHT-induced apoptosis, so the miR-370–FoxM1 pathway might be a new mechanism for HHT-induced apoptosis with a positive feedback loop between miR-370 and HHT. The regulatory mechanism in the HHT–miR-370–FoxM1 axis needs further investigatation.

We identified the role of miR-370 and FoxM1 in human CML specimens. The expression of miR-370 was lower in CML-CP and least in CML-BP patients as compared with healthy controls. In contrast, the mRNA and protein levels of FoxM1 were higher in CML-CP and highest in CML-BP patients as compared with controls. These results suggest the important function of miR-370 and FoxM1 in CML and their negative association. Recent research has showed miR-370 could be upregulated by 5-Aza-CdR, a DNA methylation inhibitor already in clinical practice [20,22]. So the combination of HHT and 5-Aza-CdR might give new insight into the treatment of leukemia. Further studies will need to confirm this hypothesis.

## Conclusions

In summary, ectopic expression of miR-370 sensitized K562 cells to HHT and partially targeted FoxM1 by inducing apoptosis. Meanwhile, HHT upregulated the level of mature miR-370. These findings might point to a way to reduce the high tolerance and toxicity of HHT and could be good news to the patients resistant to tyrosine kinase inhibitors. Therefore, a strategy combining miR-370 and HHT might be an effective clinical treatment for CML.

## Competing interests

The authors declared that they have no competing interests.

## Authors’ contributions

MZ, JZ, JJ and CC designed the study; MZ, JZ, XW, TH, YF, HS and LW performed the study; MZ, JZ, XW, QG, TH, YF, HS, LW, JJ and CC analyzed and interpreted data; CC supervised the study; and MZ, JZ, JJ and CC wrote the paper. All authors read and approved the final manuscript.

## Supplementary Material

Additional file 1: Figure S1 MTT assay of K562 cell proliferation. IC_50_ values of HHT determined by chi-square test with *P*> 0. 05, suggesting that curve fitting is good. Click here for file

Additional file 2: Figure S2 Flow cytometry of apoptosis of K562 cells with miR-370 mimics and HHT+miR-370 mimics, *P<0.05. Click here for file

Additional file 3: Figure S3 Flow cytometry of apoptosis of K562 cells with HHT+miR-370 inhibitor and HHT+NC. *P<0.05 *vs* control. Click here for file

Additional file 4: Table S1 Patient characteristics. Click here for file
