# Supplementary figures and images for: MiR-370 sensitizes chronic myeloid leukemia K562 cells to homoharringtonine by targeting Forkhead box M1
Source: J Transl Med. 2013 Oct 23;11:265. doi: 10.1186/1479-5876-11-265 (PMC4015315; doi:10.1186/1479-5876-11-265)

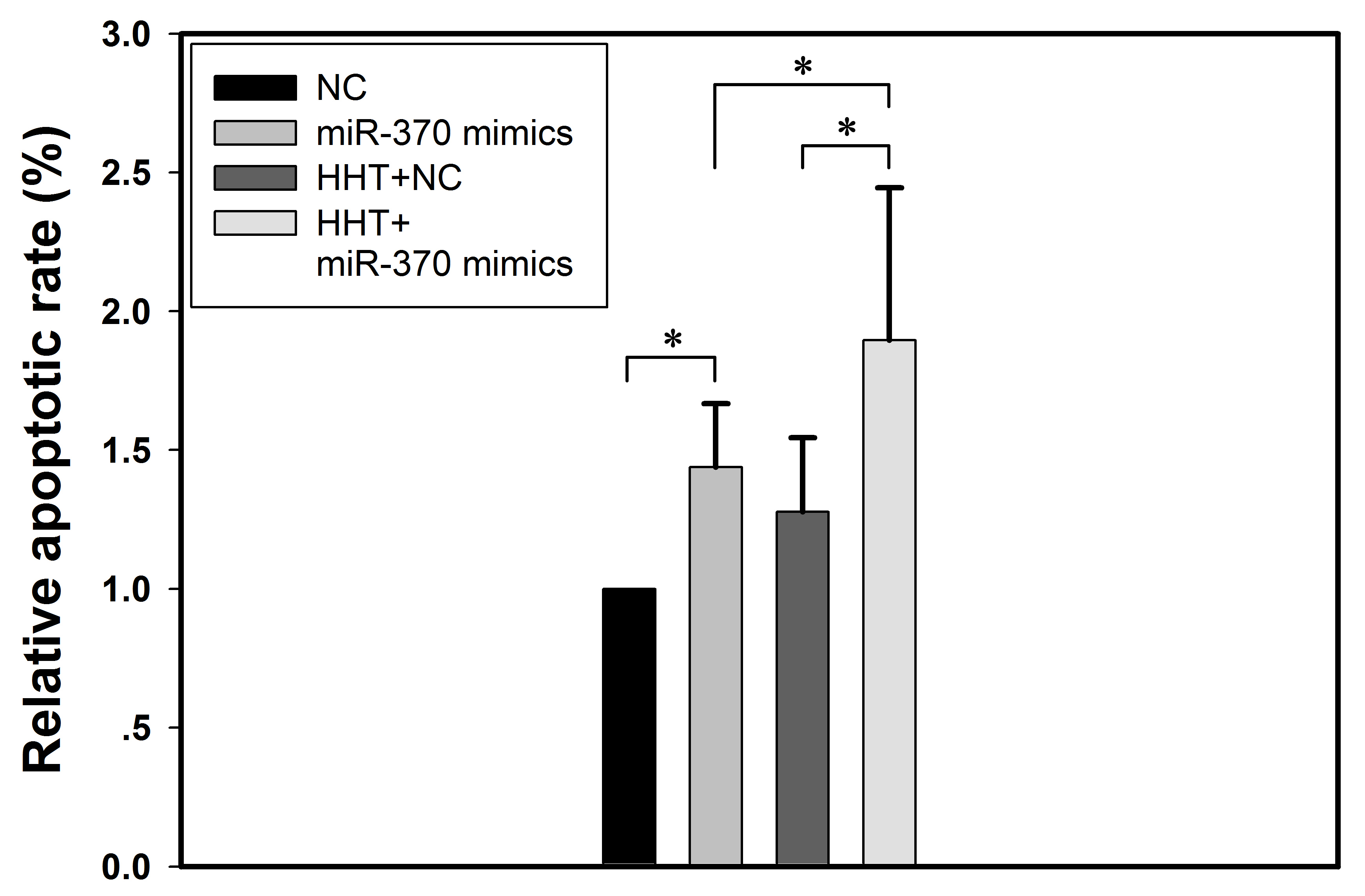

Supplement: Additional file 1: Figure S1 — MTT assay of K562 cell proliferation. IC50 values of HHT determined by chi-square test with P> 0. 05, suggesting that curve fitting is good. [file 1479-5876-11-265-S2.jpeg]

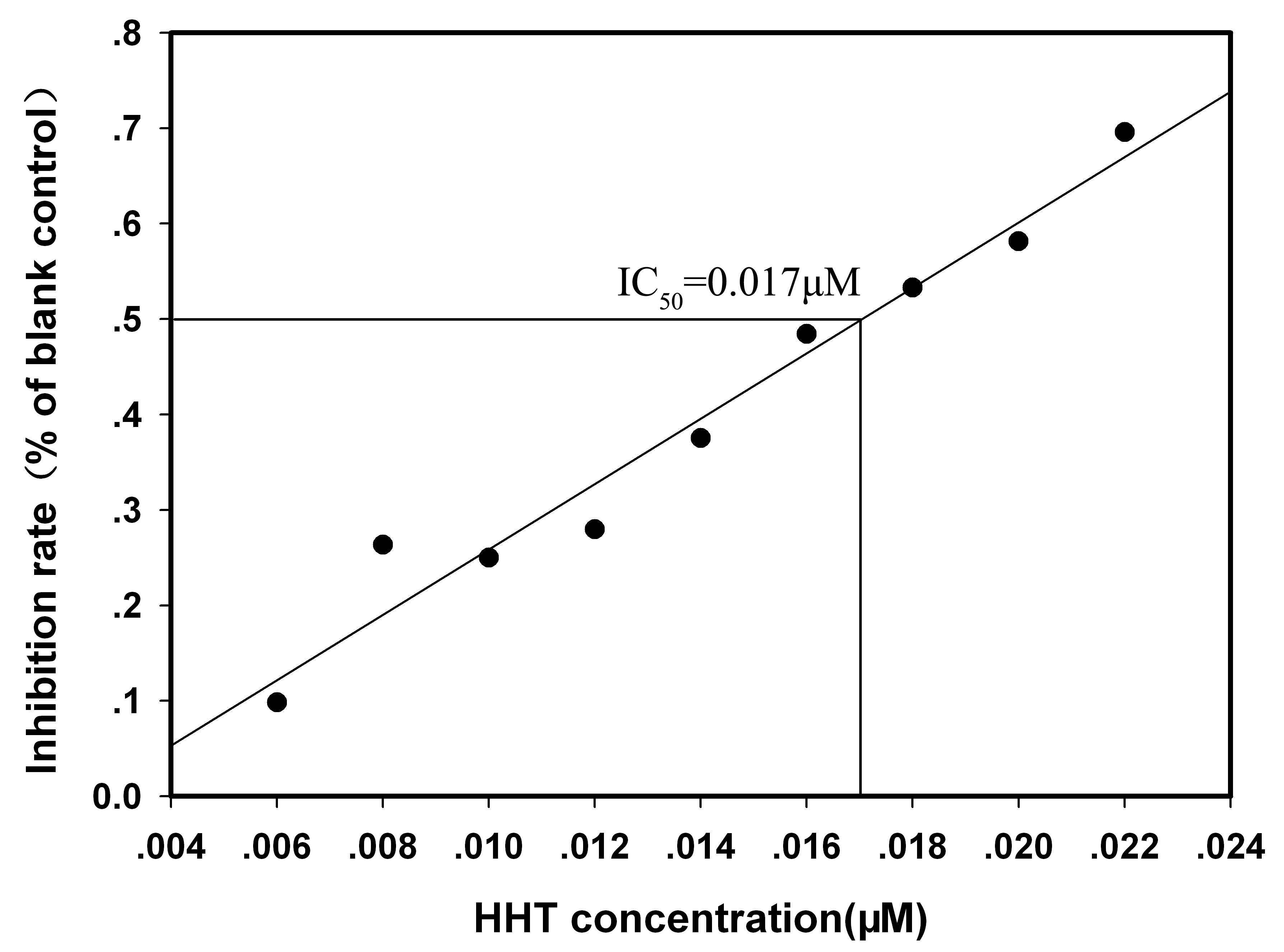

Supplement: Additional file 2: Figure S2 — Flow cytometry of apoptosis of K562 cells with miR-370 mimics and HHT+miR-370 mimics, *P<0.05. [file 1479-5876-11-265-S1.jpeg]

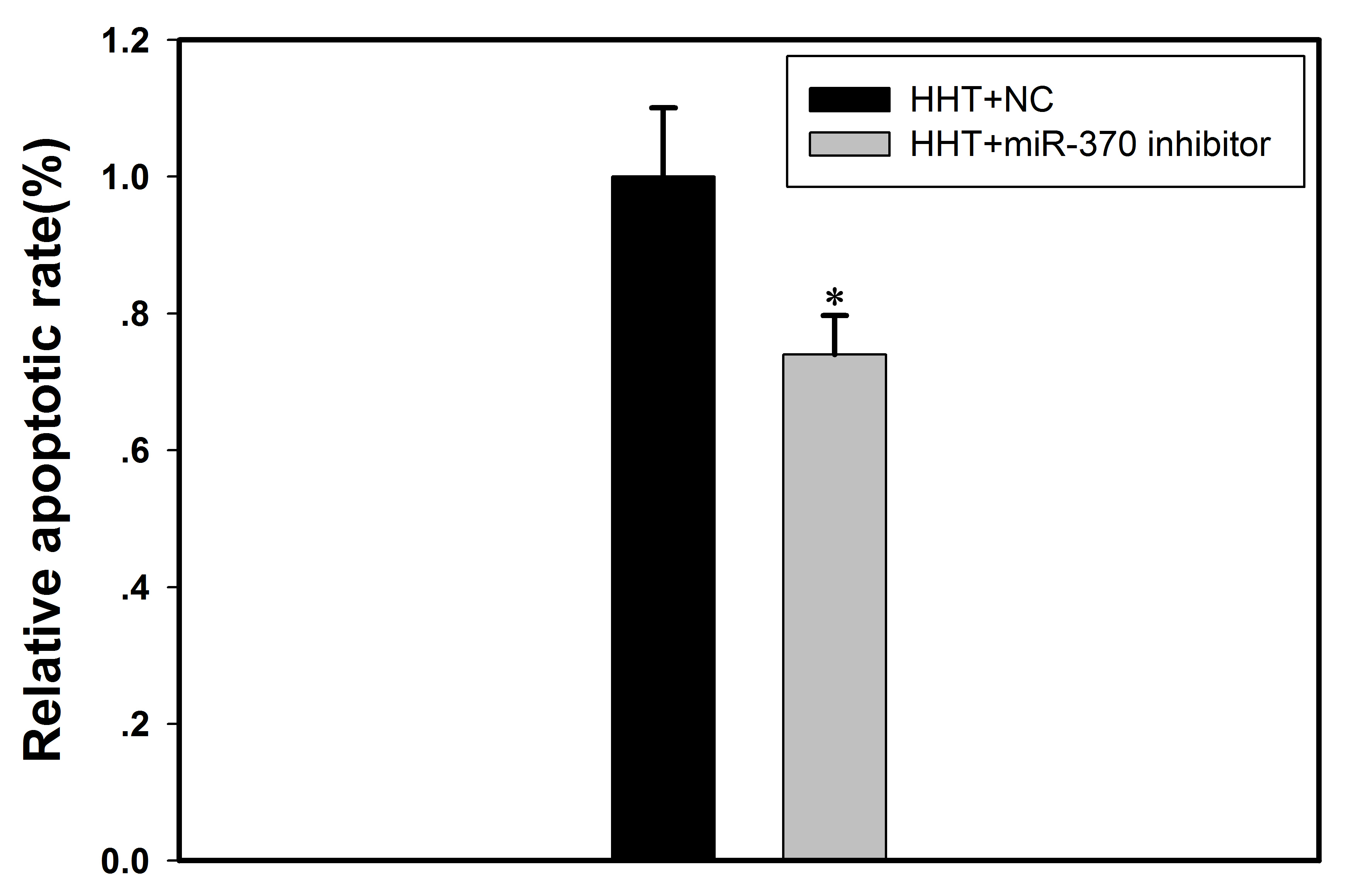

Supplement: Additional file 3: Figure S3 — Flow cytometry of apoptosis of K562 cells with HHT+miR-370 inhibitor and HHT+NC. *P<0.05 vs control. [file 1479-5876-11-265-S3.jpeg]
