# Supplementary material for: MiR-370 sensitizes chronic myeloid leukemia K562 cells to homoharringtonine by targeting Forkhead box M1
Source: J Transl Med. 2013 Oct 23;11:265. doi: 10.1186/1479-5876-11-265 (PMC4015315; doi:10.1186/1479-5876-11-265)
Supplement: Additional file 4: Table S1 — Patient characteristics. [file 1479-5876-11-265-S4.doc]

**Supplementary Table 1** Patient characteristics

| Characteristic | | CML-CP patients  (n=23) | CML-BP patients  (n=10) |
| --- | --- | --- | --- |
| Gender | Male | 18 | 5 |
|  | Female | 5 | 5 |
| Age (years) | Median | 42 | 38 |
|  | Range | 18-75 | 24-59 |
| WBC,×10^9^/L | Median | 171 | 29.5 |
|  | Range | 38.2-482 | 2.9-69.7 |
| Hemoglobin, g/L | Median | 100.9 | 82.47 |
|  | Range | 68-140 | 46-116.8 |
| Platelet count, ×10^9^/L | Median | 427.5 | 292.1 |
|  | Range | 25-1135 | 6-958 |
